# Rare hereditary nonspherocytic hemolytic anemia caused by a novel homozygous mutation, c.301C > A, (Q101K), in the AK1 gene in an Indian family

**DOI:** 10.1186/s12920-021-01038-2

**Published:** 2021-07-28

**Authors:** Rashmi Dongerdiye, Abhilasha Sampagar, Rati Devendra, Prashant Warang, Prabhakar Kedar

**Affiliations:** 1 Department of Haematogenetics, ICMR-National Institute of Immunohaematology, Indian Council of Medical Research, 13th Floor, New Multistorey Building, KEM Hospital Campus, Parel, Mumbai, 400012 India; 2 KLES Dr. Prabhakar Kore Hospital & Medical Research Centre, Belgavi, 590010 India

**Keywords:** Adenylate kinase deficiency, Enzymopathies, Prenatal diagnosis, Congenital hemolytic anemia

## Abstract

**Background:**

Adenylate kinase (AK) deficiency is a rare red cell enzymopathy associated with moderate to severe congenital nonspherocytic hemolytic anemia, along with mental and psychomotor retardation (in exceptional cases). Only ten mutations have been detected in the AK1 gene to date. In this study, we aimed to diagnose the unexplained issue of haemolytic anaemia and offer antenatal screening to the family.

**Methods:**

Genomic DNA was isolated from whole blood by a standard protocol. Targeted next-generation sequencing (t-NGS) was performed to identify pathogenic variants in the patient and control samples. A chronic villus sample was collected at 11 weeks of gestation from the mother, and molecular testing was performed. Genetic confirmation was concluded by Sanger DNA sequencing. Bioinformatics tools predicted the pathogenicity of the variant.

**Results:**

t-NGS revealed a homozygous variant (c.301C > A, p. Gln101Lys) in the *AK1* gene in the patient and heterozygosity in the fetus and parental samples. The prediction tools SIFT, Polyphen2, Provean, PMUT, Mutation taster, and Mutation Assessor, confirmed the damaging effect of the variant on the AK1 protein structure

**Conclusion:**

We have presented a novel mutation in the AK1 gene (p. Gln101Lys) associated with adenylate kinase deficiency. It is the first prenatal diagnosis of AK deficiency in India, where heterogeneity is exceptionally high.

**Supplementary Information:**

The online version contains supplementary material available at 10.1186/s12920-021-01038-2.

## Background

Adenylate kinase (AK) deficiency (OMIM 103000) is an autosomal recessive disorder associated with moderate to severe congenital hemolytic anemia, with psychomotor impairment observed in few cases [1]. The enzyme involved in this disorder is adenylate kinase type I (AK1), which catalyzes the conversion of adenine nucleotides in the presence of Mg2+ or Mn2+: Mg2++ ATP + AMP = Mg2++ ADP + ADP.

AK1 belongs to the cytosolic enzyme family (EC 2.7.4.3). The AK1 gene is located on chromosome 9 on location 9q34. 11 (NCBI Gene ID: 203) and is highly expressed mainly in tissues with a high turnover rate, such as blood, brain, and muscles[2]. According to the available literature, certain mutations in this gene resulting in a functionally inadequate enzyme; so far, only ten mutations have been reported in the *AK1* gene. [1, 3–9] (details are mentioned in Table 1).

**Table 1:** Mutations update in *AK1* gene

S. no.	Type of mutation	Nucleotide change	Amino acid change	Origin	References
1.	Missense	c.71A > G	p. Gln24Arg	Indian	Dongerdiye et al. [9]
2.	Missense	c.118G > A	p. Gly40Arg	Spanish	Corrons et al. [1]
3.	Frameshift	c.138delG	p.Glu46del	Italian	Fermo et al. [4]
4.	Missense	c.190G > A	p. Gly64Arg	Spanish	Corrons et al. [1]
5.	Missense	c.289C > T	p. Arg97Trp*	Japanese	Niizuma et al. [8]
6.	Missense	c.301C > A	p. Gln101Lys	Indian	This paper
7.	Nonsense	c.319C > T	p.Arg107Stop*	Italian	Bianchi et al. [3]
8.	Missense	c.382C > T	p.Arg128Trp	Japan	Matsuura et al. [6]
9.	Missense	c.413G > A	p.Arg138His	Indian	Dongerdiye et al. [9]
10.	Deletion	c.418_420delGAC	p.Asp140del	English	Corrons et al. [1]
11.	Missense	c.491A > G	p.Tyr164Cys	Italian	Qualtieri et al. [7]

*Mutations associated with psychomotor retardation

This study investigated the molecular basis of erythrocyte AK deficiency in an Indian family and provided prenatal diagnosis to them for subsequent pregnancy.

## Methods

### Clinical history

A 5-year-old Indian boy presented with severe neonatal jaundice and severe anemia requiring regular blood transfusions was referred to us for a complete hemolytic anemia workup. He had a hepatosplenomegaly with a liver 4 cm in size and spleen up to the umbilicus. The direct and indirect Coombs tests were negative and increased serum lactate dehydrogenase level ( 3400 U/L; reference range 140–280 U/L). The haemoglobin concentration was in the range of 5–7 gm/dl (male reference range 13–16 gm/dl). He has no history of fever and skin rashes. HPLC of the patient and parents indicated the absence of haemoglobinopathies. The Peripheral blood smear suggested dimorphic anemia with predominantly saw hypochromic, normocytic cells at the initial investigation. Bone marrow examination showed erythroid hyperplasia with megaloblasts. The biochemical test for the RBC membrane protein defect, i.e. Hereditary spherocytosis, was performed using eosin 5' maleimide by flow cytometry was within the normal range (980 MCF; reference range 900–1200 MCF). The activity of erythrocyte glucose-6-phosphate dehydrogenase, pyruvate kinase, and glucose phosphate isomerase was normal (details are given in Table 2). Presently, no developmental delay or mental retardation has been observed in the patient.

**Table 2:** Clinical and hematological data of the proband and parents

	Proband	Father	Mother	Normal range
Age/Sex	5y/M	36y/M	30y/F	–
Place of origin	Kolhapur Maharashtra			
*Hematological*				
White Blood cell (× 10^3^/µl)	9.1	8.0	9.1	4–10
Red blood cell (× 10^6^/µl)	2.14	5.03	4.53	M-4.5–5.5 F-3.8–4.8
Hemoglobin (g/dl)	6.1	14.1	11.6	M-13–17 F-12–16
Hematocrit (%)	18.5	45.7	35.7	M-45–50 F-37–45
Mean corpuscular volume (fl)	86.4	91.0	78.8	80–100
Mean corpuscular hemoglobin (Pg)	28.5	28.0	25.6	27–32
Mean corpuscular hemoglobin concentration (g/dl)	33	30.8	32.5	32–36
Platelet (× 10^3^/µl)	111	487	291	150–400
Red cell distribution width (%)	23.9	13.5	15.8	11.6–14
Retic count (%)	0.8	ND	ND	< 2.0
*Biochemical*				
Lactate dehydrogenase (U/L)	3400	ND	ND	140–280
Total bilirubin (mg/dl)	2.3	ND	ND	0.1–1.2
Direct bilirubin (mg/dl)	0.8	ND	ND	< 0.3
Hemoglobin F (%)	1.3	0.0	0.0	< 2.0
Hemoglobin A_2_ (%)	3.0	3.2	3.0	1.5–3.5
Glucose-6 phosphate dehydrogenase (IU/gHb)	5.57	6.32	5	4.0–13.0
Pyruvate kinase (IU/gHb)	9.1	10.30	8.2	8.0–14.0
Glucose phosphate isomerase (IU/gHb)	59.6	62.5	63	45–75
EMA (MCF)	980.97	956.70	946.85	900–1300
*Molecular*				
Nucleotide change	c.301C > A			
Amino acid change	p. Gln101Lys			
Zygosity	Homozygous	Heterozygous	Heterozygous	

*M* male, *F* female, *ND* not determine

### Molecular studies

We collected peripheral blood from healthy controls, patients, and parents after dually signed informed consent. DNA was extracted using a standard protocol, and a targeted next-generation sequencing (t-NGS) library was generated. We performed library preparation using Illumina's TruSeq Custom Amplicon v1.5 kit (FC 130 1001) using 250 ng genomic DNA, following the manufacturer's instructions. Samples were pooled and loaded at 20 pM on MiSeq using a v3 600 cycle reagent kit sequencing 2 × 301 paired‐end reads (Illumina, San Diego, CA, USA). The library was sequenced to mean > 80-100X coverage on the Illumina MiSeq sequencing platform. The gene panel includes red cell haemoglobinopathies, enzymopathies, membrane disorders, congenital dyserythropoietic anaemias, and bone marrow failure syndrome-related genes. Numbers of genes included in the panels with corresponding accession numbers are obtained from the Single Nucleotide Polymorphism database (dbSNP at www.nchi.nlm.nih.gov/SNP), and the Ensemble Genome Browser (www.ensembl.org) are listed in Additional file 1: Table S1). The sequences obtained were aligned to the human reference genome (GRCh37/hg19) using the BWA program and analyzed using Picard and GATK version 3.6. The clinically relevant variants were annotated with the published literature and databases such as ClinVar, OMIM, GWAS, HGMD, and SwissVar. When sequence changes were found, independent PCR products were sequenced to confirm the mutations. In support that these sequence changes were not polymorphic variations, we verified that none was reported in the 1000 Genomes, https://www.internationalgenome.org/ and Human Gene Mutation Database http://www.hgmd.cf.ac.uk/ac/index.php

### Prenatal diagnosis and detection of the familial AK deficiency causative mutation

A gynecologist conducted chronic Villus Sampling (CVS) during the 11th week of pregnancy of the mother. Genomic DNA was isolated using the standard protocol. We used the exon-specific primers mentioned by Dongerdiye 2020 et al. [9] for DNA amplification and Sanger sequencing of mother, father, and fetus samples.

### Bioinformatics analysis

The effect of the variant was studied by multiple algorithms, such as MutationTaster, https://www.mutationtaster.org Polyphen-2, https://genetics.bwh.harvard.edu/pph2 SIFT, https://sift.jcvi.org. Mutation Assessor, https://mutationassessor.org M-CAP, http://bejerano.stanford.edu/mcap/, Combined Annotation Dependent Depletion (CADD), https://cadd.gs.washington.edu/. The probability of the mutation affects protein function was evaluated. Therefore the output “low” indicates a neutral variant. For Condel, the score ranges from 0 (neutral) to 1 (damaging). The crystallographic model of recombinant human adenylate kinase (EC 2.7.4.3) was downloaded from the Protein Data Bank (www.rcsb.org/pdb/; PDB-ID: IZ83) [6]. The impact of substitution on the structure and function of the protein was studied using PyMol software (DeLano Scientific, San Carlos, CA, USA) (http://www.pymol.org/) and Swiss Protein databank viewer (https://spdbv.vital-it.ch/).

## Results

**Fig. 1:**
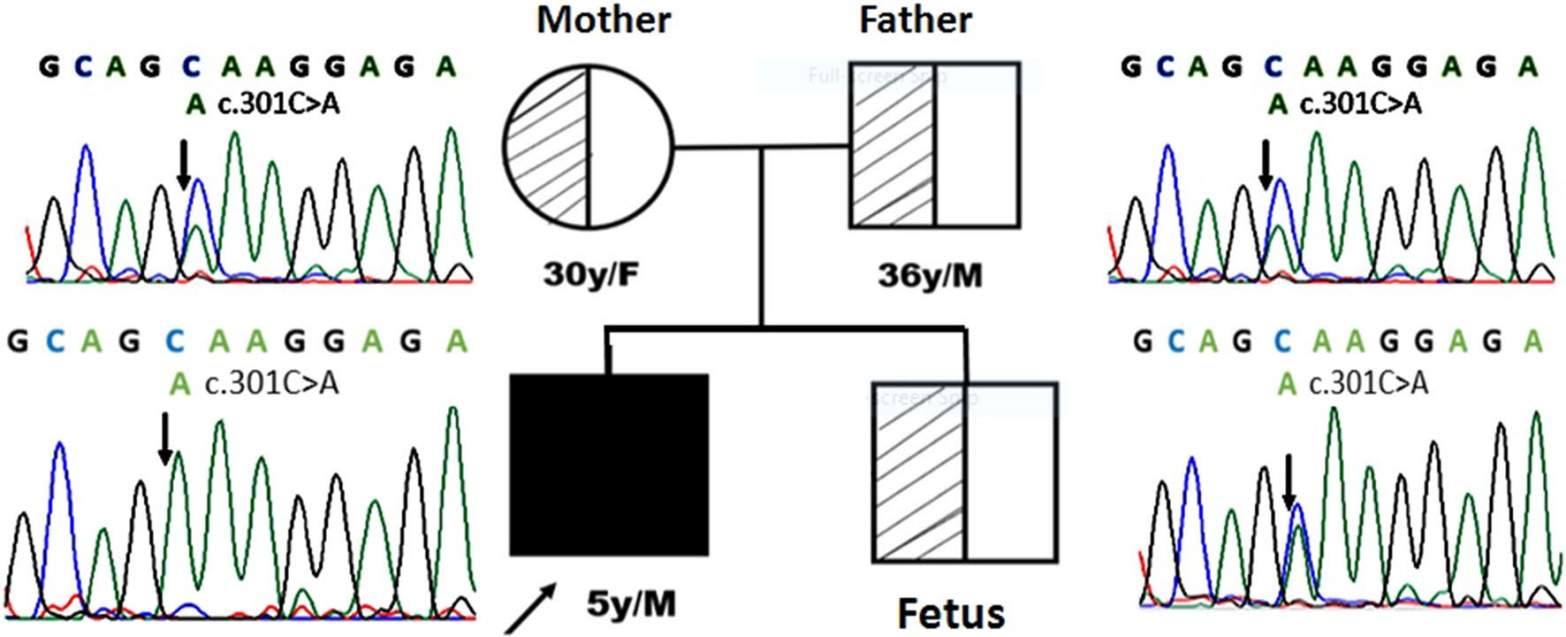
Pedigree and electropherogram of the patient and family carrying the *AK1* gene c.301C > A mutation

The complete details of the biochemical and hematological investigation of the patient and family are summarized in Table 2. Genetic analysis performed by the t-NGS panel revealed a single nucleotide substitution in exon 5 (c.301C > A) of *AK1* gene, which caused glutamine to lysine (CAA to AAA) substitution at codon 101 (p. Gln101Lys). We observed a homozygous mutation in the proband. Parents were analyzed for the c.301C > A mutation by DNA Sanger sequencing; both parents were heterozygous for the mutation (Fig. 1). The novel variant c.301C > A, p.Gln101Lys, was submitted to the ClinVar database and submitted raw data (Accession No.: PRJNA745516: https://www.ncbi.nlm.nih.gov/bioproject/PRJNA745516.) We measured AK enzyme activity in 50 healthy controls to the established normal range (reference range 297–360 IU/gHb), the proband (38.0 IU/g Hb), and parents' sample (mother 192.0 IU/gHb, father 208.0 IU/gHb). Biochemical findings correlated with molecular results.

We counseled the family for the consequences of severe enzyme deficiency. Therefore, at the time of the second pregnancy, they decided to undergo prenatal screening. The fetal DNA was screened for the complete *AK1* gene. DNA Sanger sequencing identified substitution c.301C > A at codon 101, causing a heterozygous change from glutamine to lysine. The pregnancy continued, and the normal healthy child was born after nine months and followed up for one year. There were no symptoms of anemia and jaundice.

**Fig. 2:**
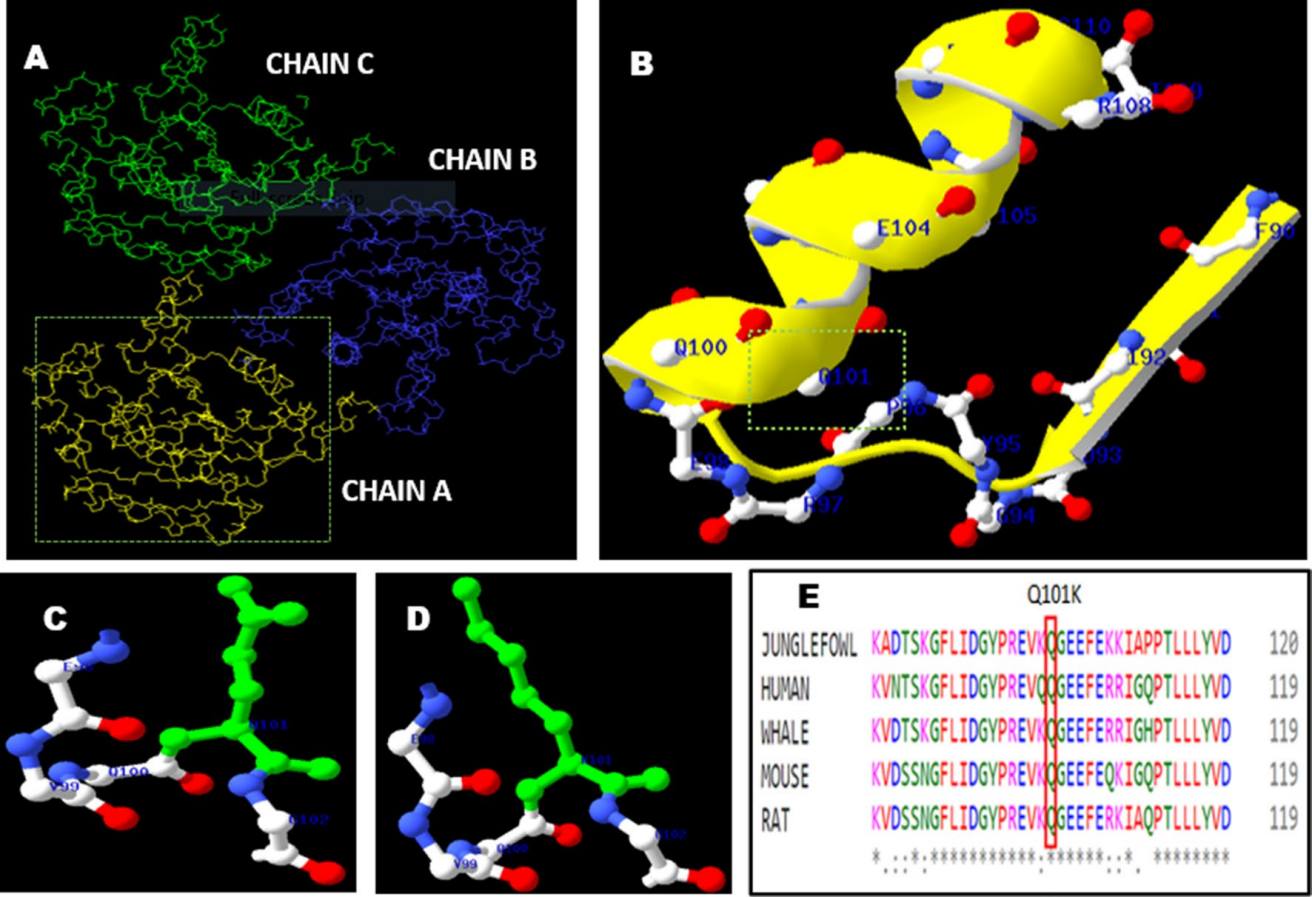
**a** Complete ribbon representation of adenylate kinase protein (PDB ID-1Z83) with chains A, B, and C. **b** Secondary structure of the protein (PDB ID-1Z83) showing the amino acid residue at position 101 (Q101). **c** Wild type amino acid residue glutamine 101 (Q101). **d** Mutant type amino acid residue Lysine101 (K101). **e** The residue (Q) at position 101 of AK-1 is highly conserved across species

The AK1 protein (PDB ID-1Z83) consists of three chains, A, B, and C, spanning 194 residues. Each chain consists of one large central "CORE" domain and two small peripheral domains, the NMP binding and the LID domains. Upon ATP binding, the LID domain closes over the phosphoryl transfer site. The amino acid residue position Q101 is an important AMP binding site and 39,44,138,149 residues. Any changes at these AMP binding sites possibly hamper the catalytic cycle of the enzyme. Figure 2a shows a complete ribbon representation of the protein (PDB ID-1Z83) with chains A, B, and C of the AK1 enzyme, along with an insight into the Q101 position helical structure (Fig. 2b) and amino acid change from wild type (glutamine) to mutant type (lysine) (Fig. 2c, d). Multiple sequence alignment confirmed that the amino acid glutamine at 101 positions is conserved across species (Fig. 2e). Most bioinformatic prediction tools demonstrate the harmful effect of the amino acid change from glutamine to lysine (Table 3 summarizes the prediction results).

**Table 3:** Bioinformatical prediction data

Tool	Score	Prediction
Polyphen-2	1.00	Damaging
PROVEAN	− 3.969	Deleterious
Mutation taster	–	Disease-causing
Mutation assessor	4.965	HIGH
MUTPRED2	0.882	–
SIFT	0.001	Damaging
PMUT	94%	Disease
CADD	17.33	Likely benign
M-CAP	0.458	Possibly pathogenic
REVEL	0.5	Likely disease-causing

## Discussion

In the present study, an Indian patient was diagnosed with rare red cell adenylate kinase deficiency. A novel homozygous (p. Gln101Lys) mutation in the *AK1* gene was detected using a disease-targeted NGS panel. Previously, we reported the first case of AK deficiency from India, caused by compound heterozygous (c.71A > G and c.413G > A) mutations in the *AK1* gene [9]. Clinically, these patients have incidents of chronic hemolytic anemia but no evidence of mental retardation or psychomotor impairment. Similar to other erythrocyte enzyme deficiencies, the choice of treatment depends upon the severity of the disease. Splenectomy may be recommended for severe transfusion-dependent AK deficient patients [14]. During the study, we excluded all the possible causes of hemolytic anemia, including RBC membrane defects. A recent review on red cell membrane defects mentioned that the flow‐cytometric osmotic fragility test is a new gold standard method for diagnosing HS, HE, and DHS in combination with eosin‐5'‐maleimide testing [13].

In India, only a few research groups have incorporated NGS-based genetic analysis for the routine diagnosis of RBC enzymopathy [9, 10]. However, custom NGS panels or whole-genome sequencing are widely used in various other laboratories to diagnose hemolytic anemia. The use of next-generation sequencing allows the identification of new causative genes, and polygenic conditions, and genetic factors that modify the disease severity of hereditary anemias. Disease-targeted gene panels often have higher diagnostic rates than those of exome sequencing or genome sequencing and are designed to maximize coverage, sensitivity, and specificity for the included genes [11]. The only drawback of using a custom NGS panel is that it involves a limited number of genes. Therefore, a continuous update is required for the best results. We have achieved an approximately 80% diagnostic yield using our custom panel [12]. However, these results may vary depending upon the number of patients, their clinical history, and phenotype-genotype correlations.

We identified three missense substitutions in the A*K1* gene, Gln24Arg, Gln101Lys, and Arg138His, in two unrelated Indian families. These mutations could lead to dysfunction of the enzyme molecule by hampering AMP binding capacity. This study focuses on providing prenatal diagnosis to the family and gives accurate genetic advice. The fetal DNA was heterozygous for the substitution c.301C > A; p.Gln101Lys and advised to continue the pregnancy. Next-generation sequencing has many advantages, as it is cost-effective and gives high yield and speed. In contrast, there are certain limitations of NGS that significantly impact the accuracy of the results. The t-NGS panel has proven precise for our study, but its application may vary from lab to lab.

## Conclusion

In conclusion, the targeted NGS panel identified a novel causative mutation in the *AK1* gene in a 5-year-old male child with severe transfusion-dependent haemolytic anaemia. Identification of the pathogenic mutation helped us to offer a prenatal diagnosis in this family. This study also re-emphasizes the importance of NGS in diagnosing unexplained haemolytic anaemia in severe patients.

## Supplementary Information

**Additional file 1**. Gene List for Targated NGS Panel.

## Data Availability

All the data generated or analyzed in this study are included in this manuscript. All the gene sequences were retrieved from Ensemble Genome Browser (www.ensembl.org). We received Accession numbers for novel mutation in *AK1* gene in exon 5 (c.301C > A, p. Gln101Lys) submitted on ClinVar submission Portal (Accession No: VCV001162194.2 and Variation ID: 1162194). Reference datasets used in this study is human reference genome (GRCh37/hg19), (https://www.ncbi.nlm.nih.gov/clinvar/variation/1162194/). The datasets generated and analyzed during the current study are available in the NCBI Sequence Read Archive Database (Accession No.: PRJNA745516: https://www.ncbi.nlm.nih.gov/bioproject/PRJNA745516. The web links of the relevant datasets were as follows: hg19 http://genome.ucsc.edu/, 1000 Genomes project (http://www.1000genomes.org/), dbSNP (http://www.ncbi.nlm.nih.gov/snp), gnomAD (https://gnomad.broadinstitute.org/about), ClinVar (https://www.ncbi.nlm.nih.gov/clinvar/), and OMIM (http://omim.org).

## Abbreviations

ADP: Adenosine diphosphate
AK: Adenylate kinase
AMP: Adenosine monophosphate
ATP: Adenosine triphosphate
BWA: Burrows-wheeler alignment
CVS: Chronic villus sample
DNA: Deoxyribonucleic acid
EDTA: Ethylenediaminetetraacetic acid
EMA: Eosin 5 maleimide
G6PD: Glucose-6-phosphate-dehydrogenase
GATK: Genomeanalysistoolkit
GPI: Glucose phosphate isomerase
GWA: Genome wide association study
HB: Haemoglobin
HCT: Hematocrit
HGMD: Human genome mutation database
HPLC: HIGH: performance liquid chromatography
LDH: Lactate dehydrogenase
MCF: Mean channel fluorescence
MCH: Mean corpuscular haemoglobin
MCHC: Mean corpuscular haemoglobin concentration
MCV: Mean corpuscular volume
NGS: Next-generation sequencing
NMP: Nucleoside monophosphate
OMIM: Online mendelian inheritance in man
PDB: Protein data bank
PDB: Protein databank
PK: Pyruvate kinase
PLT: Platelet
PROVEAN: Protein variation effect analyzer
RBC: Red blood cell
RDW: Red cell distribution width
SIFT: Sorting intolerant from tolerant
t-NGS: Targeted-next-generation sequencing
WBC: White blood cell

## References

[1] Corrons J-LV (2003). Red cell adenylate kinase deficiency: a molecular study of 3 new mutations (118G>A, 190G>A, and GAC deletion) associated with hereditary nonspherocytic hemolytic anemia. Blood.

[2] Noma T (2005). Dynamics of nucleotide metabolism as a supporter of life phenomena. J Med Investig.

[3] Bianchi P (1999). A case of complete adenylate kinase deficiency due to a nonsense mutation in the AK-1 gene (Arg 107> Stop, CGA>TGA) associated with chronic hemolytic anemia. Br J Haematol.

[4] Fermo E (2004). A new variant of adenylate kinase (delG138) associated with severe hemolytic anemia. Blood Cells Mol Dis.

[5] Miwa S (1983). Red cell adenylate kinase deficiency associated with hereditary nonspherocytic hemolytic anemia: clinical and biochemical studies. Am J Hematol.

[6] Matsuura S (1989). Human adenylate kinase deficiency associated with hemolytic anemia. A single base substitution affecting the solubility and catalytic activity of the cytosolic adenylate kinase. J Biol Chem.

[7] Qualtieri A (1997). Severe erythrocyte adenylate kinase deficiency due to homozygous A → G substitution at codon 164 of human AK1 gene associated with chronic haemolytic anaemia. Br J Hematol.

[8] Niizuma H (2017). Splenectomy resolves hemolytic anemia caused by adenylate kinase deficiency. Pediatr Int.

[9] Dongerdiye R (2019). Red cell adenylate kinase deficiency in India: identification of two novel missense mutations (c.71A>G and c.413G>A). J Clin Pathol.

[10] Jamwal M (2020). Next-generation sequencing-based diagnosis of unexplained inherited hemolytic anemias reveals wide genetic and phenotypic heterogeneity. J Mol Diagn.

[11] Russo R (2020). Genetics and genomics approaches for diagnosis and research into hereditary anemias. Front Physiol.

[12] Kedar PS (2019). Study of pathophysiology and molecular characterization of congenital anemia in India using targeted next-generation sequencing approach. Int J Hematol.

[13] Iolascon A (2019). Advances in understanding the pathogenesis of red cell membrane disorders. Br J Haematol.

[14] Iolascon A (2017). Recommendations for splenectomy in hereditary hemolytic anemias. Haematologica.

