# Supplementary material for: Rare hereditary nonspherocytic hemolytic anemia caused by a novel homozygous mutation, c.301C > A, (Q101K), in the AK1 gene in an Indian family
Source: BMC Med Genomics. 2021 Jul 28;14:191. doi: 10.1186/s12920-021-01038-2 (PMC8317388; doi:10.1186/s12920-021-01038-2)
Supplement: Supplementary file 1 — Additional file 1. Gene List for Targated NGS Panel. [file 12920_2021_1038_MOESM1_ESM.docx]

**Supplementary Table S1-:** Targeted NGS panel for the analysis of 80 genes responsible for rare congenital anaemias.

| Sr. No | Gene name | Description | Strand Direction | Accession Number  (*) |
| --- | --- | --- | --- | --- |
|  | *ABCB7* | ATP Binding Cassette Subfamily B Member 7 | - | NM_004299 |
|  | *ABCG8* | ATP Binding Cassette Subfamily B Member 8 | + | NM_022437 |
|  | *ADA* | Adenosine Deaminase | - | NM_000022 |
|  | *ADD1* | Alpha Adducins | + | NM_014189 |
|  | *AK-1* | Adenylate Kinase-*1* | - | NM_000476 |
|  | *ALAS2* | Aminolevulinate Synthase 2 | - | NM_000032 |
|  | *ALDOA* | Aldolase | + | NM_000034 |
|  | *ANK1* | Ankyrin | - | NM_001142446 |
|  | *BPGM* | Bisphosphoglycerate Mutase | + | NM_199186 |
|  | *C15orf41* | Chromosome 15 open reading frame 41 | + | NM_001130010 |
|  | *CDAN1* | Codanin 1 | - | NM_138477 |
|  | *CECR1* | Cat Eye Syndrome Chromosome Region, Candidate 1 | - | NM_001282228 |
|  | *DKC1* | Dyskeratosis Congenita 1, Dyskerin | + | NM_001363 |
|  | *ENO1* | Enolase 1 | - | NM_001428 |
|  | *EPB41* | Erythrocyte Membrane Protein Band 4.1 | + | NM_001166005 |
|  | *EPB42* | Erythrocyte Membrane Protein Band 4.2 | - | NM_000119 |
|  | *EPB72* | Stomatin (EPB72)-Like 1 | - | NM_004809 |
|  | *FECH* | Ferrochelatase | - | NM_000140 |
|  | *G6PD* | Glucose 6 Phosphate Dehydrogenase | - | NM_000402 |
|  | *GATA1* | Gata Binding Protein 1 | + | NM_002049 |
|  | *GCLC* | Gamma-glutamyl cysteine synthetase deficiency | - | NM_001498 |
|  | *GPI* | Glucose Phosphate Isomerase | + | NM_001184722 |
|  | *GPX1* | Glutathione peroxidase | - | NM_000581 |
|  | *GSR* | Glutathione-Disulfide Reductase | - | NM_000637 |
|  | *GSS* | Glutathione Synthetase | - | NM_000178 |
|  | *GYPA* | Glycophorin A | - | NM_002099 |
|  | *GYPC* | Glycophorin C | + | NM_002101 |
|  | *HBA1* | Hemoglobin Subunit Alpha 1 | + | NM_000558 |
|  | *HBB* | Hemoglobin Subunit Beta | - | NM_000518 |
|  | *HK1* | Hexokinase | + | NM_000188 |
|  | *HMOX1D* | Heme Oxygenase (Decycling) 1 | + | NM_002133 |
|  | *HSPA9* | Heat shock 70KDa Protein 9 | - | NM_004134 |
|  | *KCNN4* | potassium intermediate/small conductance calcium-activated channel, subfamily N, member 4 | - | NM_002250 |
|  | *KIF23* | Kinesin Family Member 23 | + | NM_138555 |
|  | *KLF1* | Kruppel Like Factor 1 | - | NM_006563 |
|  | *LPIN2* | Lipin 2 | - | NM_014646 |
|  | *NADH-MR(CYB5R3)* | Cytochrome B5 reductase 3 | - | NM_001171660 |
|  | *NT5C3A* | 5'-Nucleotidase, Cytosolic IIIA | - | NM_016489 |
|  | *PFK-M* | Phosphofructokinase, Muscle | + | NM_001166686 |
|  | *PGD* | 6-Phosphogluconate dehydrogenase | + | NM_002631 |
|  | *PGK1* | Phosphoglycerate Kinase 1 | + | NM_000291 |
|  | *PIEZO1* | Piezo type mechanosensitive ion channel component 1 | - | NM_001142864 |
|  | *PIEZO2* | Piezo type mechanosensitive ion channel component 2 | - | NM_022068 |
|  | *PIGA* | Phosphatidylinositol Glycan Anchor Biosynthesis, Class A | - | NM_002641 |
|  | *PIGT* | Phosphatidylinositol Glycan Anchor Biosynthesis, Class T | + | NM_015937 |
|  | *PKLR* | Pyruvate kinase | - | NM_000298 |
|  | *PUS1* | Pseudouridylate Synthase 1 | + | NM_025215 |
|  | *RhAG* | Rh Associated Glycoprotein | - | NM_000324 |
|  | *RPL11* | Ribosomal Protein L11 | + | NM_000975 |
|  | *RPL26* | Ribosomal Protein L26 | - | NM_000987 |
|  | *RPL27* | Ribosomal Protein L27 | + | NM_000988 |
|  | *RPL35A* | Ribosomal Protein L35A | + | NM_000996 |
|  | *RPL5* | Ribosomal Protein L5 | + | NM_000969 |
|  | *RPL9* | Ribosomal Protein L9 | - | NM_001024921 |
|  | *RPS10* | Ribosomal Protein S10 | - | NM_001014 |
|  | *RPS17* | Ribosomal Protein S17 | - | NM_001021 |
|  | *RPS19* | Ribosomal Protein S19 | + | NM_001022 |
|  | *RPS24* | Ribosomal Protein S24 | + | NM_001142285 |
|  | *RPS24* | Ribosomal Protein S24 | + | NM_001142285 |
|  | *RPS26* | Ribosomal Protein S26 | + | NM_001029 |
|  | *RPS27* | Ribosomal Protein S27 | + | NM_001030 |
|  | *RPS7* | Ribosomal Protein S7 | + | NM_001011 |
|  | *RTEL1* | Regulator Of Telomere Elongation Helicase 1 | + | NM_001283009 |
|  | *SBDS* | Shwachman-Bodian-Diamond Syndrome | - | NM_016038 |
|  | *SEC23B* | Sec23 Homolog B, Coat Complex II Component | + | NM_006363 |
|  | *SLC11A2* | Solute Carrier Family 11 (Proton-Coupled Divalent Metal Ion Transporter), Member 2 | - | NM_001174125 |
|  | *SLC19A2* | Solute Carrier Family 19, Member 2 | - | NM_006996 |
|  | *SLC25A38* | Solute Carrier Family 25, Member 38 | + | NM_017875 |
|  | *SLC2A1* | Solute Carrier Family 2 (Facilitated Glucose Transporter), Member 1 | - | NM_006516 |
|  | *SLC4A1* | Solute Carrier Family 4 Member 1 | - | NM_000342 |
|  | *SPTA1* | Spectrin Alpha, Erythrocytic 1 | - | NM_003126 |
|  | *SPTB* | Spectrin Beta, Erythrocytic | - | NM_001024858 |
|  | *STEAP3* | STEAP Family Member 3, Metalloreductase | + | NM_182915 |
|  | *TERC* | Telomerase RNA Component | - | NR_001566 |
|  | *TINF2* | TERF1 (TRF1)-Interacting Nuclear Factor 2 | - | NM_001099274 |
|  | *TMPRSS6* | Transmembrane Serine Protease 6 | - | NM_153609 |
|  | *TPI1* | Triosephosphate Isomerase 1 | + | NM_001159287 |
|  | *TRNT1* | tRNA Nucleotidyl Transferase, CCA-Adding, 1 | + | NM_182916 |
|  | *UGT1A1* | UDP Glucuronosyltransferase 1 Family, Polypeptide A1 | + | NM_000463 |
|  | *YARS2* | Tyrosine-tRNA Synthetase | - | NM_001040436 |

*Accession number provided in the last column is obtained from the Single Nucleotide Polymorphism database (dbSNP at [www.nchi.nlm.nih.gov/SNP](http://www.nchi.nlm.nih.gov/SNP)) or in the Ensemble Genome Browser ([www.ensembl.org](http://www.ensembl.org)).
